# Children With Cerebral Palsy Playing With Mainstream Robotic Toys: Playfulness and Environmental Supportiveness

**DOI:** 10.3389/fpsyg.2018.01814

**Published:** 2018-09-26

**Authors:** Daniela Bulgarelli, Nicole Bianquin, Serenella Besio, Paola Molina

**Affiliations:** ^1^Department of Psychology, Università degli Studi di Torino, Turin, Italy; ^2^Department of Social and Human Sciences, Università della Valle d’Aosta, Aosta, Italy; ^3^Department of Social and Human Sciences, Università degli Studi di Bergamo, Bergamo, Italy

**Keywords:** physical impairment, Test of Playfulness, Test of Environmental Supportiveness, play, scaffolding

## Abstract

**Purpose:** Play is a right for every child and has a key role in child development. Play can be analyzed according to the construct of playfulness, which is the child’s disposition to play. Children with cerebral palsy (CP) show difficulties in play and can also experience lower playfulness scores when compared to matched typically developing children. This paper analyses play and playfulness in children with CP using mainstream robotic toys with supporting adult play partners.

**Methodology:** Five mainstream robotic toys were selected and used in play situations with six children with CP interacting with two adult partners. The play situations were coded through the Test of Playfulness (ToP) and the Test of Environmental Supportiveness (ToES), to analyze the role of robotic toys, adult partners and environment in supporting play and playfulness in children with CP.

**Results:** The children obtained high ToP scores, showing that they were intrinsically motivated to be engaged in the play situations. The ToP scores weakly correlated with the ToES scores. To discuss this result, different features of each robot, the role of adults as scaffolder, and the space characteristics in supporting play are presented and discussed.

**Conclusion:** This research field is new: to our knowledge, in the literature only one study focused on the use of one type of mainstream robotic toy to support the playfulness of children with CP. The parallel use of the ToP and the ToES was crucial to observe the complexity of the play situations and the role of playmates and toys during the play process. The role of the adult as play scaffolder has been important to mediate between the child with CP and the environment, toys included: the adult should be strongly aware of this role to better support the child in being in charge of the play situation. Further research is needed.

## Play and Playfulness in Children with Cerebral Palsy

Play is a complex construct that can be approached from different points of view. According to Garvey (1990), it is a range of voluntary activities that are internally motivated and usually associated with enjoyment and pleasure. Authors in the field have differently underlined the role of play in child development and, according to their epistemological approaches; have stressed in some cases the cognitive dimension, originally proposed by Piaget (1962), or the social one, at first formulated by Parten (1932). Recently, the COST Action TD1309 “LUDI – Play for Children with Disabilities" proposed a developmental classification of play based on both dimensions and their main theoretical typologies: practice, symbolic, constructive and rule play with respect to the cognitive domain, and solitary, parallel, associative and cooperative play with respect to the social one (for a wider description, see Bulgarelli and Bianquin, 2017).

Within the LUDI Action a distinction between play and play-like activities has been adopted: in play-like activities, play is not performed “for the sake of play” itself, but it is used as a means to reach other goals, such as learning or enriching abilities (Visalberghi, 1958; Besio, 2017). Play can be also analyzed according to the construct of playfulness that is the child’s “disposition to play” (Barnett, 1991) and is composed of four elements (Skärd and Bundy, 2008).

(1) Source of Motivation – the players engage in play simply because they want to; some aspects of the activity itself drive the children’s involvement: the source of motivation is intrinsic.
(2) Perception of Control – the players feel they are in charge of their own actions and at least of some aspects of the activity outcomes.
(3) Suspension of Reality – the players are not bound by unnecessary constraints of reality and they can choose how close the activity should be to objective reality.
(4) Framing – it refers to the player’s ability to give and interpret social cues about how to interact with the play partners.

Playfulness partly depends on the players’ characteristics and on the environmental features, that can favor play or interfere with it (Bronson and Bundy, 2001). Children with disabilities may face difficulties in play, because of their physical and/or cognitive impairments, and because contexts, objects and toys may not be accessible and usable. Moreover, compared to typically developing children, in everyday life children with disabilities are more likely to experience play-like activities rather than play “for the sake of play,” because very often they are mainly involved in rehabilitation and school activities. Given that play has a crucial role in the individual physical, cognitive, affective and social development (Piaget, 1962; Vygotsky, 1976), the limitations in play can be detrimental for children with disabilities, and can limit their quality of life (Besio, 2017). Moreover, the Convention on the Rights of the Child (United Nations Human Rights, 1989) and the Convention of the Rights of Persons with Disabilities (United Nations Human Rights, 2006) underline that play is an unalienable right of the child and, as such, it has to be supported both at individual and societal levels.

Cerebral Palsy (CP) is a neurological disorder caused by a non-progressive brain injury or abnormal brain development that occurs before birth, during birth, or immediately after birth. CP affects body movement, muscle control, muscle coordination, muscle tone, reflex, posture and balance. It can also impact fine motor skills, gross motor skills and oral motor functioning. It may be associated to impairments in cognitive development, communication and sensorial perception (Rosenbaum et al., 2007). Children with CP show difficulties in any type of play and can also experience lower playfulness scores when compared to matched typically developing children (Okimoto et al., 2000; Smidt and Cress, 2004; Hamm, 2006; Childress, 2011; Pfeifer et al., 2011; Besio and Amelina, 2017). Differently from these results, Harkness and Bundy (2001) found that children with CP scored on the Test of Playfulness (ToP) similarly as gender and aged-matched typically developing children. Nevertheless, the authors acknowledged that in the two groups, the play conditions were different in terms of environmental supportiveness: children with CP were mainly observed at home with similar aged siblings (i.e., a situation supporting playfulness), and typically developing children were mainly observed at playgrounds/parks with peers.

## Mainstream Robotic Toys to Support Play and Playfulness

In the light of the World Health Organization, 2007 International Classification of Functioning–Version Children and Youth (ICF–CY), the promotion of play and playfulness for children with disabilities can be approached by intervening on two areas: on personal factors, for instance sustaining motivation (Tatla et al., 2013), or on environmental factors, i.e., by improving parents’ conceptualization of play and teaching them how to effectively deal with play interactions (Okimoto et al., 2000; Graham et al., 2014), or by acting on the environment, by adapting toys and play situations (Hsieh, 2008).

In the literature, examples of robotic prototypes designed to support the play of children with disabilities showed critical aspects. Low playful attractiveness and need for substantial support from adults on one hand (IROMEC: Besio and Carnesecchi, 2016; van den Heuvel et al., 2016) and unsolved accessibility issues on the other hand (PALMIBER: Ceres et al., 2005; Raya et al., 2015).

Robotic toys might allow to overcome some of these issues. They offer different play scenarios and interaction modalities and their supportiveness for playfulness is worth to be studied in-depth. Moreover, they can be easily purchasable and inexpensive, thus facilitating a possible impact on the everyday life of a vast majority of children and families. To our knowledge, in the literature only one study involved the use of mainstream robotic toys. Ríos-Rincón et al. (2016) observed playfulness in four children with CP playing with their mothers in three conditions: (a) baseline sessions, (b) intervention sessions in which an adapted Lego robot was introduced and children were trained to move it and make it carry objects, and (c) follow-up sessions. The play sessions with the robotic toy were characterized by higher playfulness levels.

## The Role of the Adult as Scaffolder

The concept of scaffolding (Wood et al., 1976) refers to the tutorial process an expert partner plays to assist and foster someone who is less skilled during the learning process. According to the authors, adults and children are usually involved in “instructional relationships” (Wood et al., 1976, p. 89) and successful scaffolders put in place several supportive behaviors: they keep the children motivated, focus their attention on essential aspects of the task, divide it into manageable sub-tasks, and model solutions. A key feature of the scaffolding process is its temporary nature: the adult’s support should progressively fade, because scaffolding is meant to guide the less skilled partners along their zone of proximal development (Vygotsky, 1978) and to enable them to become autonomous (Hammond and Gibbons, 2005). When playing together, adults or peers can act as scaffolder toward the children, to support play and playfulness (Bianquin, 2018).

## The GioDi Project

The GioDi (Gioco per la Disabilità – Play for Disability) Project aimed at testing the recreational potential of mainstream robotic toys in supporting playfulness when used by children with CP. The project involved seven children playing with five robotic toys–in different separated sessions–and expert adults. The project’s first results showed that all the robots supported children’s playfulness. Nevertheless, the role of the expert adults interacting with the child was crucial: they acted as an assistive play companion and a scaffolding partner (Besio et al., 2016a,b). Toy accessibility improvements were also proposed and realized. In some cases, the toy interface was integrated with other existing technologies; in other cases, the toy was concretely modified – substituting the standard controller with another one adapted to the child’s hand and grasp – or some parts of the toy or their interfaces were substituted (for more details, see: Veronese et al., 2016).

## The Current Study

This paper reports in-depth additional results of the GioDi Project. It aims at addressing more precisely the role of the expert adult and the role of the toy in supporting playfulness during the play. According to previous results reported in the literature (Bronson and Bundy, 2001; Besio et al., 2016a,b; Ríos-Rincón et al., 2016), we expected the children’s playfulness to be positively associated with two environmental features: the adults’ and the mainstream robotic toys supportiveness.

## Materials and Methods

### Sample

Six Italian children (five boys and one girl) were selected according to two criteria: diagnosis of CP^1^ and age between 7 and 11 years (see **Table 1**). The children’s mean age was 9.15 years (*SD* = 1.47). To uniformly describe the children’s characteristics, two tools were used. The Gross Motor Function Classification System (GMFCS; Palisano et al., 2008) was used to assess the children’s physical ability; the Vineland Adaptive Behavior Scales-II (VABS-II, Italian version: Balboni et al., 2016) were administered to the children’s parents and were used to assess the children’s abilities in everyday life contexts. Three children had certified comorbid conditions.

**Table 1:** Description of the sample.

*Child*	*Age range in years*	*Certified comorbidities*	*GMFCS level*	*VABS-II IQ*
C_2	9–10	Dysarthria and anarthria	V	34
		Epilepsy		
		Conduct disorder		
C_3	9–10	Dysarthria and anarthria	V	26
C_4	8–9		V	39
C_5	7–8	Dysarthria and anarthria	V	61
C_6	9–10		IV	73
C_7	10–11		IV	21


According to GMFCS, for children between 6 and 12 years, level IV means that children need physical assistance or powered mobility in most life environments while, at home, they can move along short distances by rolling, creeping or crawling. Level V means that children show limitations in arm and leg movements; they need manual wheelchair to move in all environments, and have difficulties in maintaining antigravity head and trunk postures. For a more detailed description of the levels, see Palisano et al. (2008). The VABS-II allow to calculate IQ scores, based on a norm group with an average score of 100 and a standard deviation of 15. Thus, a score of 100 means that the child’s functioning is normal with respect to his/her age; typical IQs range between 70 and 130, whereas atypical IQs are below 70 or above 130. Parents gave their written consent for their children to participate in the study; children agreed to play, showed to be involved in the activities with the researchers and did not ask to interrupt the sessions.

### Robotic Toys

Five mainstream robotic toys have been selected through a search on the Internet; five criteria guided the choice:

(a) the toy should allow different types of play (practice, symbolic, constructive, and rule; Bulgarelli and Bianquin, 2017);
(b) the toy should show different functions (move, sound, etc.);
(c) the toy input systems should be modifiable through technological intervention according to the child’s needs; the possibility to modify the input system was inferred from the technical data sheet provided by the manufacturers;
(d) the toys should be different from one another with respect to aesthetic features and configurations;
(e) the toy should have an affordable price (i.e., max 200€).

Air Swimmer, Cubelets, Dash and Dot, Edison and Zoomer were finally selected^2^. Air Swimmer is a helium-inflated blimp, about 60 cm long, shaped like a shark or a clown fish; it is capable of moving in the air by flapping the rear fin and adjusting its balance; commands are sent through an infrared remote controller, including two rocker buttons. Cubelets are a set of strong plastic cubes, each containing a special characteristic: a sensor, an actuator, a power supply, or another special element; they can be connected to each other thanks to magnetic docks and can exchange information and power. Dash and Dot are a couple of robots – one of which is able to move in the environment – with colored lights, sounds, and humanlike mimics; they can be used together or separately, thanks to specific apps for smart phones and tablets. Edison looks like a small orange parallelepiped with two wheels. Its sensors make it possible for it to react to sounds, light, proximity, and to follow lines. This toy also has several actuators (lights, speaker, and motors) and is programmable either via a programming language, or by reacting to a bar code that activates one out of the six pre-loaded different games. Zoomer is a robotic pet dog that can move autonomously in the environment (it can roll, play dead, sing, pee, etc.). The actions can be triggered by pressing a button, or by issuing a specific vocal command in English. A wider description of the toys has been presented in Veronese et al. (2016).

### Procedure

Each child carried out three play sessions together with two adults, a psychologist expert on play interaction and an engineer expert on robotic toys. In each session, two or three toys were individually presented. Session 1 was dedicated to Air Swimmer and Cubelets, session 2 to Edison and Zoomer, and session 3 to Dash and Dot, which required more time to explore all its functioning. If the child was not interested in a toy, then the adults proposed another one. The child had at least 3 min to autonomously explore each toy. After, the adults showed the children all the characteristic of each robot at a time and encouraged them to play with it. The children’s reactions were observed, to verify if they were aware of the undergoing play situation, and to change the play scenarios so that the activity might be tuned with the children’s interests and capabilities. The children’s proposals were supported, reinforced and enlarged with new possibilities. All these interventions were inspired by a scaffolding approach.

The play sessions were held in a laboratory and were fully video-recorded. Each child passed three play sessions, one per week in about 1 month; one child (C_6) who was not interested in playing with Cubelets passed two play sessions. The sessions lasted between 45 and 55 min each and the time devoted to each robot varied, according to the interest showed by the child.

### Tools and Measures

Two tools were used by three observers (two observers per each tool) to code at least 10 min of each play session: the ToP and the Test of Environmental Supportiveness (ToES). The ToP (Skärd and Bundy, 2008) was used to evaluate playfulness. It has shown validity, reliability and to be adequate for use with children with CP (Bundy et al., 2001). The ToP is made of 21 items; each item is rated from 0 to 3 according to three dimensions: extent, intensity and/or skilfulness, when applicable. To score the child’s playfulness, the item scores are inserted into the ToP Keyform, that takes into account the relative difficulty of each item and allows to obtain a unique measure for the total playfulness, that can vary from -3 to +3. The observer #1 coded the ToP and the observer #2 independently scored the 17% of the play sessions; the inter-rater agreement was good (Cohen’s *K* = 0.76).

The ToES (Bundy, 1999) addresses the role of different variables supporting play: caregivers; peer, older, younger playmates; natural/fabricated objects; sensory environment; configuration, safety and space accessibility. The ToES was designed to be used simultaneously with the ToP to assess play through a “person-in-environment” approach (Bronson and Bundy, 2001). The ToES have also shown validity, reliability and to be adequate for use with children with and without disabilities (Bronson and Bundy, 2001; Hamm, 2006). The ToEs is composed by 17 items to be scored as follows: -2 (strongly interferes), -1 (slightly interferes), 1 (slightly favors), and +2 (strongly favors). In the current study, an average score of the three items dedicated to the older playmates (in our case, expert adults) was calculated, to check the possible effect of the adults’ interaction on the total playfulness scores. The observer #3 coded the ToES; the observer #2 independently scored the 20% of the play sessions; the inter-rater agreement was moderate (Cohen’s *K* = 0.60).

Twenty-five observations have been coded. In three situations, it was not possible to code the ToP and ToES scores, due to various technical reasons (in one case, the video failed in being recorded; in other cases, the toy was broken); in other two situations, the children dedicated too little time to play with Air Swimmer and Cubelets (see **Table 2**).

**Table 2:** ToP total scores per child/robotic toy.

*Child*	*Air swimmer*	*Cubelets*	*Dash and dot*	*Edison*	*Zoomer*	*Mean score (SD)*	*GMFCS level*	*VABS-II IQ*
C_2	0.40	1.90	0.60	2.10	2.00	1.40 (0.83)	V	34
C_3	1.90	1.80	1.90	1.90	1.50	1.80 (0.17)	V	26
C_4	NA	0.00	0.90	0.20	1.00	0.53 (0.50)	V	39
C_5	RP	2.30	0.80	0.90	0.90	1.23 (0.72)	V	61
C_6	NA	RP	2.20	2.10	2.10	2.13 (0.06)	IV	73
C_7	1.30	2.50	NA	0.90	1.30	1.50 (0.69)	IV	21
Mean score (SD)	1.20 (0.75)	1.70 (0.99)	1.28 (0.72)	1.35 (0.79)	1.47 (0.50)			


*NA, not applicable; RP, refuse to play.*

### Data Analysis

Descriptive analyses were run on both the ToP and the ToES scores: average scores and standard deviations are reported. Pearson’s product-moment correlations were used to measure the association between the ToP and ToES scores, and between these tools and the GMFCS and the VABS. The absolute value of the Pearson’s correlation represents the strengths of the relationship between two variables and it can be used to interpret the association effect size (Cohen, 1997): 0.10 represent a weak association, 0.30 a moderate association and 0.50 a strong association.

## Results

The ToP scores varied between 0.00 and 2.50 (*M* = 1.42, *SD* = 0.71). The scores were higher than 1.49 in 13 sessions (52%), and were lower than 0.50 in 3 sessions (12%); according to these data, the children were involved in playful and fun activities with the mainstream robotic toys. The ToP scores varied according to the child and the toy involved in the play situations: Cubelets and Zoomer obtained higher average ToP scores (see **Table 2**).

The ToES scores varied between 1.18 and 2.00 (*M* = 1.80, *SD* = 0.20). The scores were higher than 1.49 in 24 sessions (96%). Thus, the playmates, the objects and the physical environment positively supported the play activities (see **Table 3**).

**Table 3:** ToES total scores per child/robotic toy.

*Child*	*Air swimmer*	*Cubelets*	*Dash and dot*	*Edison*	*Zoomer*	*Mean score (SD)*	*GMFCS level*	*VABS-II IQ*
C_2	2.00	1.91	2.00	2.00	1.64	1,40 (0.83)	V	34
C_3	2.00	2.00	1.75	1.82	2.00	1.80 (0.17)	V	26
C_4	NA	1.50	1.91	1.91	1.91	0.53 (0.50)	V	39
C_5	RP	1.73	2.00	1.50	1.82	1.22 (0.72)	V	61
C_6	NA	RP	1.82	1.75	1.63	2.13 (0.69)	IV	73
C_7	1.71	1.18	NA	1.82	1.64	1.50 (0.69)	IV	21
Mean score (SD)	2.00 (0.17)	1.66 (0.33)	1.87 (0.11)	1.76 (0.16)	1.75 (0.14)			


The ToP and the ToES scores were correlated with the children’s measures of gross motor functions (GMFCS) and adaptive behavior (VABS-II). The GMFCS did not significantly correlate with the ToP but the effect size was moderate (Pearson’s *R* = -0.317, *p* = 0.123); it significantly correlated with the ToES (Pearson’s *R* = 0.463, *p* = 0.020). The VABS-II did not correlate either with the ToP nor with the ToES and the effect sizes were weak (respectively, Pearson’s *R* = 0.084, *p* = 0.689 and Pearson’s *R* = -0.059, *p* = 0.778). Since the correlation between the total ToP scores and the total ToES scores was not significant (Pearson’s *r* = -0.212, *p* = 0.309), we investigated if the ToP scores correlated with some specific environmental aspects explored through the ToES: the adult playmates, the toys and the space characteristics.

Three ToES items address the role of older playmates, i.e., the adults. The play sessions involved two adults: the first one was expert in child play and interaction, the second one was skilled in robotic toys. The adult #1 was always scored as a strongly supportive playmate: she adequately responded to the child’s cues, supporting the play logic, waiting for the child’s response and contributing to maintain the play flow; she also contributed to the interaction by giving clear cues and supporting the continuation of the play frame or a change to it; and she participated as an equal player, being involved, contributing with ideas but without being manipulative or bossy. The adult #2 was scored as strongly supportive in the 80% of the sessions, and slightly supportive in the remaining 20%. Given that the supportiveness of the adult #1 was a numeric constant, it was not possible to calculate the correlation between that score and the ToP. The correlation between the ToP scores and the average scores of adult #2 was not significant (Pearson’s *r* = -0.292, *p* = 0.211).

One ToES item is dedicated to the supportiveness of natural or fabricated objects during the play activities, in this case, the robotic toys. This item was used as the specific index of supportiveness of each robot. In the ToES, toys facilitate play when they support the children in their efforts to fulfill motivation, allow modification of challenges and engender feelings to do something with it. **Table 4** reports the average “supportiveness of object” scores each toy obtained. The correlation between the ToP scores and the toys supportiveness was not significant (Pearson’s *r* = 0.076, *p* = 0.717).

**Table 4:** Average scores (SD) of the ToP and the ToES item “Natural/fabricated objects support activity of player” by robotic toy.

*Toy*	*No. of Sessions*	*Object supportiveness score (SD)*	*ToP scores (SD)*
Air Swimmer	3	1.67 (0.58)	1.20 (0.75)
Cubelets	5	1.00 (1.23)	1.70 (0.99)
Dash	5	1.60 (0.55)	1.28 (0.72)
Edison	5	1.33 (0.52)	1.35 (0.79)
Zoomer	6	0.00 (1.55)	1.47 (0.50)


## Discussion

The GioDi project aimed at testing playfulness in children with CP and at addressing the role of expert adult play partners and mainstream robotic toys in supporting playfulness. In general, the children obtained high playfulness scores (**Table 2**): according to the behaviors that the ToP allows to observe, the children were intrinsically motivated to be engaged in the play situations; they were in control of the play scenarios because they could choose how to play with the robots, and had fun; the interactions with the adult partners was positive and aiming at carrying on the play situation. Some of the children faced difficulties with respect to suspension of reality, and it was not possible to score the ToP items related to this aspect of playfulness. The high ToP scores could be also linked to the novelty of the situation. In fact, the parents reported that the children were happy and excited to come to the lab to play with the robots and the adults and, during the days between the meetings, some children expressly asked to go back to the lab. With respect to the average ToP scores per robot (**Table 2**), the children appeared to mostly enjoy Cubeletes (1.70) and Zoomer (1.47); Edison, Dash and Dot and Air Swimmer were attributed lower scores (1.35, 1.28, and 1.20, respectively). Nevertheless, each child obtained the highest playfulness score with a specific toy and none of the robots was the most playful to everybody.

Playfulness is the child’s disposition to play (Barnett, 1991) and it can be linked to other child’s characteristics. In our study, ToP scores were positively associated with gross motor abilities – in fact, the absolute *R*-value was moderate – and were not correlated with children’s adaptive behavior. Moreover, playfulness also depends on the overall supportiveness the environment provides during the play session. The current study aimed at observing the environmental supportiveness and to specifically focus on the role of two contextual aspects: the adult interacting with the child and the type of toy, i.e., mainstream robots. To do this, the ToES had been coded and Pearson’s correlation analysis of some ToES single items had been run as well. According to previous results reported in the literature (Bronson and Bundy, 2001; Besio et al., 2016a,b; Ríos-Rincón et al., 2016), the children’s playfulness was expected to be positively associated with both the adults’ and the mainstream robotic toys supportiveness. In our study the ToP total scores did not significantly correlate with the total scores related to the environmental supportiveness, measured through the ToES. Nevertheless, the *r* absolute value could be interpreted as effect size: the association between children’s playfulness and environmental supportiveness was weak. This was an unexpected result and some possible explanations are envisaged. The low variability of the ToES total scores could have determined the lack of correlation. The play sessions were run in a controlled context and this could have affected the ToES variability. In fact, the play partners were adults trained to adequately interact with the children and support their play activities and the toys were selected to be attractive and interesting.

The analysis of some single items composing the ToES seemed to confirm this hypothesis. The adult partners were mainly scored as strongly supportive. For this reason, it was not possible to correlate the score of adult #1, whose marks were constantly high; the ToES subscores of adult #2 also did not significantly correlate with the ToP total scores but the effect size of this negative association was moderate: the more playful the child, the less supportive the adult. Apparently, the adults intervene especially when the child seemed to need support to maintain a fun play experience. In fact, the adults played the crucial role of scaffolders, both on the relational/educational dimension (being emotionally responsive, accepting and developing the play scenarios the children proposed, supporting them, etc.) and on the technical/contextual one (fixing technical problems, helping the child in using or manipulating the robots, adapting the space configuration when possible, etc.). Effectively supporting the children’s playfulness, the adults respected their level of autonomy, gradually trying to increase it. Their role was played according to the scaffolding construct and it had been shaped as physical guide, modeling or verbal suggestion. Thus, the adults performed different levels of control over the play situation, attuning to the capacity the child was showing with that particular toy, and the children had different possibilities to interiorize and elaborate the adult’s proposals (Besio et al., 2016b).

The robots also supported playfulness. Air Swimmer and Dash and Dot obtained the highest average ToES scores (1.67 and 1.60, respectively), i.e., they mainly favored play in a strong way; Edison and Cubelets scores (1.33 and 1.00, respectively) showed that they slightly favored play, whereas Zoomer’s score (0.00) showed that it also interfered with play. This finding is coherent with the results observed by Ríos-Rincón et al. (2016) and it is particularly important since mainstream robots were used in this study: they can be considered as an important opportunity for children with physical limitations to experience play for the sake of play.

To better understand the differences among the robot ToES scores, it is useful to analyze their shape and configuration, and to take into consideration that the toys never came alone, but were accompanied by expert adults. A description of the accessibility and usability issues of the robots is reported also in the study by Veronese et al. (2016).

Air Swimmer was the robot to better support the play activities, probably because it did not require refined motor abilities to be manipulated and its design promptly suggested a fun play scenario: it was mainly used as a scary shark swimming in the air to be punched away. Thus, the children were playful: they were highly involved and amused by the situation; they shared glances, smiles and vocalizations with the play partners and, even if they could not control the robot autonomously through the controller, they could interact with it in a satisfactory way through their hand and arm movements while punching it away.

Edison could move thanks to the vibrations created by clapping hands or beating on the surface it was running on. This allowed the children with the most severe fine motor impairment to voluntary act on the toy in order to obtain the result they were expecting: making the robot move. This feeling of control over the object strongly supported playfulness. In the case of Edison, the adults’ support was mainly technical and addressed to activate the robot, by quickly pressing – as required – two small buttons in a fixed sequence, and to help the children customize their own Edison, by sticking Lego bricks on the top of the robot.

The other robots required more controlled fine motor abilities to be manipulated. For instance, the Cubelets magnetic modules could be assembled in different ways to create objects that move following lights, avoiding obstacles, etc., but sticking them together required a sophisticated matching between modules and precise oculo-motor coordination. In addition, they tended to separate when children touched them too roughly: without the technical support by the adults, children could not have played with them successfully.

Dash and Dot nicely supported the play activities too. It enabled different play scenarios involving pictures and sounds but it presented some accessibility issues: the robot needed to be used through a devoted software application via tablet or smartphone, that could not be effectively used by children with CP, and a technical support by the adults was necessary.

In the initial part of the play activity, Zoomer proved to be an enjoyable toy, because its shape, movements and sounds were fun and made it look like a real dog. Nevertheless, after some minutes, the play experience was also frustrating. Zoomer was activated through a precise sequence of touch and phonologically precise vocal commands, that was challenging for the children who had difficulties in controlling their upper limb movements and could not speak precisely (or could not speak at all). Moreover, as a sort of real dog, the robot was designed to be trained by a personal master and it got acquainted with one person’s voice: as a matter of fact, it ended up answering to one of the expert adult’s voice only. A button on Zoomer’s back could be used to produce random behaviors: in some case, this allowed the children to autonomously make it move, but they missed the fun aspect of controlling the dog, by giving it a command and seeing it “obeying.” All these reasons affected Zoomer’s ToES scores, which were the lowest ones.

Thus, a deeper analysis of the toy supportiveness showed that the mainstream robots were an interesting solution to better promote play and playfulness in children with CP, but the play partner still had a crucial role to overcome the accessibility and usability issues. The design of these robots was not meant for all children and it was often difficult to adapt them to each child’s specific needs; first possible adaptations of the toy input systems, through technological interventions, have been proposed by the research group (Veronese et al., 2016). On one hand, awareness about the importance of the “design for all” (Norman, 2013) needs to be raised among the toy designers and the toys companies. On the other hand, educators should be trained to better modulate their interaction with the child during the play activity, to support play for the sake of play and playfulness, i.e., to foster the child’s internal control, intrinsic motivation and possibility to suspend reality to access symbolic play. In fact, these are two necessary steps to better promote the right to play for every child and to foster playfulness itself, that is linked to the child’s quality of life.

Not surprisingly, the total ToES scores correlated with the children’s gross motor function scores: the more impaired the motor function, the higher the ToES scores. This result stresses the necessity to intervene on the environment to better support children’s play and playfulness.

A limit of the current research refers to the small sample size and to the possible selection bias due to the voluntary participation of the families; nevertheless, the clinical conditions of children with severe CP made the recruitment of large and representative samples difficult. Some limitations were also related to the accessibility and usability of the robots. For instance, the Dash and Dot apps were designed for English-speakers only, and this created a barrier for the Italian children involved in the project. The ToP and the ToES are rating scales and a critical aspect in the use of these tools was the difficulty in scoring the items, because a clear distinction between each level of item was not specified; this had an effect on the inter-rater reliability scores, which was moderate.

## Conclusion

This study showed that children with CP were playfully involved with five mainstream robotic toys; during the sessions, the adult playmates scaffolded the child’s interaction with the toys and almost all the robots (four out of five) supported playfulness as well. Thus, mainstream robotic toys are an interesting play proposal for children with CP. Contrary to the study of Ríos-Rincón et al. (2016), in this case the children had just one occasion to play with each robot and no training was organized so that they may learn to better control the toy. Thus, further research could deepen if a long lasting use of these mainstream robots may support playfulness or, on the opposite, make the children get tired of them.

The parallel use of the ToP and the ToES was crucial to observe the complexity of the play situations and the role of playmates and toys during the play process. The role of the adult as play scaffolder has been important to mediate between the child with CP and the environment, toys included: the adult should be strongly aware of this role to better support the child in being in charge of the play situation. The scaffolding approach allows to modulate the adults’ intervention, that will progressively fade out, thus allowing the child’s autonomous play. For this reason, future research will focus on systematizing a model about the role of the adult as scaffolder to support play for the sake of play. Moreover, a replication of the study in everyday environment is needed to check any potential differences due to specific contexts. Finally, a deeper analysis of the spatial configuration and characteristics could also be interesting to observe the impact of the play space on playfulness. A further research area could be focused on the development of customized adaptations for the toy accessibility and usability and on testing the efficacy of these adaptations in better supporting playfulness.

## Ethics Statement

This study was carried out in accordance with the ethical recommendations of the Ethical Code of the Italian Psychologists Association. The protocol was approved by the Bioethics Committee of the Università degli Studi di Torino. All subjects gave their written informed consent in accordance to the Declaration of Helsinki.

## Author Contributions

All authors designed the study. DB and SB contributed to the data collection. DB and NB performed the data analysis. DB wrote the paper. NB, SB, and PM revised it.

## Conflict of Interest Statement

The authors declare that the research was conducted in the absence of any commercial or financial relationships that could be construed as a potential conflict of interest.

## Footnotes

1 One additional child diagnosed with a degenerative muscle disease (not better specified) was observed but not included in this study.

2 Toy websites: Air Swimmer: http://airswimmers.com; Cubeletes: www.modrobotics.com/cubelets; Dash and Dot: https://www.makewonder.com/dash; Edison: https://meetedison.com; Zoomer: https://www.zoomerpup.com/.

## References

[B1] Balboni G. Belacchi C. Bonichini S. Coscarelli A. (2016). *Vineland II.* Firenze: Organizzazioni Speciali.

[B2] Barnett L. A. (1991). The playful child: measurement of a disposition to play. *Play Cult.* 4 51–74.

[B3] Besio S. (2017). “The need for play for the sake of play,” in *Play Development in Children with Disabilities*, eds Besio S. Bulgarelli D. Stancheva Popkostadinova V. (Warsaw: De Gruyter Open), 9–52.

[B4] Besio S. Amelina N. (2017). “Play in Children with Physical Impairment,” in *Play Development in Children with Disabilities*, eds Besio S. Bulgarelli D. Stancheva Popkostadinova V. (Warsaw: De Gruyter Open), 120–136.

[B5] Besio S. Bonarini A. Bulgarelli D. Carnesecchi M. Riva C. Veronese F. (2016a). “Is play easier for children with physical impairment with mainstream robots? Accessibility issues and playfulness,” in *Proceeding of the Conference Universal Learning Design*, eds Peňáz, P. Hanousková M. Ondra S. (Brno: Masaryk University), 97–107.

[B6] Besio S. Bonarini A. Lynch H. Molina P. Veronese F. Bulgarelli D. (2016b). “Mainstream robotic toys and children with physical impairment: what about playfulness?,” in *Proceeding of the 7th International Conference on Software Development and Technologies for Enhancing Accessibility and Fighting Info-exclusion*, (New York, NY: The Association for Computing Machinery), 232–239. 10.1145/3019943.3019974

[B7] Besio S. Carnesecchi M. (2016). “Caregivers’ support for children with motor impairments in robot-mediated play contexts. a case study for the investigation of adult/child relationship in supporting play development,” in *Proceeding of the Conference Universal Learning Design*, eds Peňáz, P. Hanousková M. Ondra S. (Brno: Masaryk University), 109–114.

[B8] Bianquin N. (2018). “How can I, as an adult, facilitate play?,” in *Guidelines for Supporting Children with Disabilities’ Play: Methodologies, Tools, and Contexts*, eds Encarnação P. Ray-Kaeser S. Bianquin N. (Warsaw: De Gruyter Open), 50–58. 10.1515/9783110613445-009

[B9] Bronson M. R. Bundy A. C. (2001). A correlational study of a test of playfulness and a test of environmental supportiveness for play. *Occup. Ther. J. Res.* 21 241–259. 10.1177/153944920102100403

[B10] Bulgarelli D. Bianquin N. (2017). “Conceptual review of play,” in *Play Development in Children with Disabilities*, eds Besio S. Bulgarelli D. Stancheva Popkostadinova V. (Warsaw: De Gruyter Open), 58–70.

[B11] Bundy A. C. (1999). *Test of Environmental Supportiveness.* Fort Collins, CO: Colorado State University.

[B12] Bundy A. C. Nelson L. Metzger M. Bingaman K. (2001). Validity and reliability of a test of playfulness. *Occup. Ther. J. Res.* 21 276–292. 10.1177/153944920102100405

[B13] Ceres R. Pons J. L. Calderon L. Azevedo L. (2005). “A robot vehicle for disabled children,” in *Proceedings of the IEEE Engineering in Medicine ad Biology Magazine* Vol. 24 (Monterey, CA: IEEE), 55–63. 10.1109/MEMB.2005.1549731 16382806

[B14] Childress D. C. (2011). Play behaviors of parents and their young children with disabilities. *Topics Early Child. Spec. Educ.* 31 112–120. 10.1177/0271121410390526

[B15] Cohen J. (1997). “The earth is round (p < .05),” in *What If There Were No Significance Tests?*, eds Harlow L. L. Mulaik S. A. Steiger J. H. (New York, NY: Psychology Press), 21–36.

[B16] Garvey C. (1990). *Play.* Cambridge, MA: Harvard University Press.

[B17] Graham N. Truman J. Holgate H. (2014). An exploratory study: expanding the concept of play for children with severe cerebral palsy. *Br. J. Occup. Ther.* 77 358–365. 10.4276/030802214X14044755581781 25871596

[B18] Hamm E. M. (2006). Playfulness and the environmental support of play in children with and without developmental disabilities. *OTJR* 26 88–96. 10.1177/153944920602600302

[B19] Hammond J. Gibbons P. (2005). What is scaffolding. *Teach. Voices* 8 8–16.

[B20] Harkness L. Bundy A. C. (2001). The test of playfulness and children with physical disabilities. *Occup. Ther. J. Res.* 21 73–89. 10.1177/153944920102100203

[B21] Hsieh H. C. (2008). Effects of ordinary and adaptive toys on pre-school children with developmental disabilities. *Res. Dev. Disabil.* 29 459–466. 10.1016/j.ridd.2007.08.004 17936580

[B22] Norman D. A. (2013). *The Design of Everyday Things: Revised and Expanded Edition.* New York, NY: Basic Books.

[B23] Okimoto A. M. Bundy A. C. Hanzlik J. (2000). Playfulness in children with and without disability: measurement and intervention. *Am. J. Occup. Ther.* 54 73–82. 10.5014/ajot.54.1.73 10686630

[B24] Palisano R. J. Rosenbaum P. Bartlett D. Livingston M. H. (2008). Content validity of the expanded and revised gross motor function classification system. *Dev. Med. Child Neurol.* 50 744–750. 10.1111/j.1469-8749.2008.03089.x 18834387

[B25] Parten M. (1932). Social play among preschool children. *J. Abnorm. Soc. Psychol.* 27 243–269. 10.1037/h0074524

[B26] Pfeifer L. I. Mota Pacciulio A. Abrão dos Santos C. Lício dos Santos J. Stagnitti K. E. (2011). Pretend play of children with cerebral palsy, physical and occupational therapy. *Pediatrics* 31 390–402. 10.3109/01942638.2011.572149 21574911

[B27] Piaget J. (1962). *Play, Dreams and Imitation in Childhood.* New York, NY: Norton.

[B28] Raya R. Rocon E. Urendes E. Velasco M. A. Clemotte A. Ceres R. (2015). “Assistive robots for physical and cognitive rehabilitation in cerebral palsy,” in *Intelligent Assistive Robots. Springer Tracts in Advanced Robotics* Vol. 106 eds Mohammed S. Moreno J. C. Kong K. Amirat Y. (Berlin: Springer International Publishing), 133–156. 10.1007/978-3-319-12922-8_5

[B29] Ríos-Rincón A. M. Adams K. Magill-Evans J. Cook A. (2016). Playfulness in children with limited motor abilities when using a robot. *Phys. Occup. Ther. Paediatr.* 36 232–246. 10.3109/01942638.2015.1076559 26566226

[B30] Rosenbaum P. Paneth N. Leviton A. Goldstein M. Bax M. Damiano D. (2007). A report: the definition and classification of cerebral palsy April 2006. *Dev. Med. Child Neurol.* 49 8–14. 10.1111/j.1469-8749.2007.tb12610.x 17370477

[B31] Skärd G. Bundy A. C. (2008). “Test of Playfulness,” in *Play in Occupational Therapy for Children*, eds Parham L. D. Fazio L. S. (Amsterdam, NL: Elsevier), 71–93. 10.1016/B978-032302954-4.10004-2

[B32] Smidt M. L. Cress C. J. (2004). Mastery behaviors during social and object play in toddlers with physical impairments. *Educ. Train. Dev. Disabil.* 39 141–152.

[B33] Tatla S. K. Sauve K. Virji-Babul N. Holsti L. Butler C. Loos H. F. M. (2013). Evidence for outcomes of motivational rehabilitation interventions for children and adolescents with cerebral palsy: an American Academy for Cerebral Palsy and Developmental Medicine systematic review. *Dev. Med. Child Neurol.* 55 593–601. 10.1111/dmcn.12147 23550896

[B34] United Nations Human Rights (1989). *Convention on the Rights of the Child.* Available at: http://www.ohchr.org/EN/ProfessionalInterest/Pages/CRC.aspx

[B35] United Nations Human Rights (2006). *Convention of the Rights of Persons with Disabilities.* Available at: https://www.un.org/development/desa/disabilities/convention-on-the-rights-of-persons-with-disabilities.html

[B36] van den Heuvel R. Lexis M. Jansens R. de Witte L. (2016). “Professionals view on IROMEC play sessions for children with severe physical disabilities,” in *Proceeding of the Conference Universal Learning Design*, eds Peňáz P. Hanousková M. Ondra S. (Brno: Masaryk University), 115–199.

[B37] Veronese F. Bulgarelli D. Besio S. Bianquin N. Bonarini A. (2016). “Off-the-shelf, robotic toys and physically impaired children: an analysis and suggested improvements,” in *Proceeding of the 7th International Conference on Software Development and Technologies for Enhancing Accessibility and Fighting Info-exclusion*, (New York, NY: The Association for Computing Machinery), 232–239. 10.1145/3019943.3019977

[B38] Visalberghi A. (1958). *Esperienza e Valutazione [Experience and Evaluation].* Torino: Taylor.

[B39] Vygotsky L. S. (1976). “Play and its role in the mental development of the child,” in *Play. Its Role in Development and Evolution*, eds Bruner J. S. Jolly A. Silva K. (New York, NY: Basic Book), 537–554.

[B40] Vygotsky L. S. (1978). *Mind in society: The Development of Higher Psychological Processes.* Cambridge, MA: Harvard University Press.

[B41] Wood D. Bruner J. S. Ross G. (1976). The role of tutoring in problem solving. *J. Child Psychol. Psychiatry* 17 89–100. 10.1111/j.1469-7610.1976.tb00381.x 932126

[B42] World Health Organization (2007). *International Classification of Functioning, Disability, and Health: Children, and Youth Version: ICF-CY.* Geneva: World Health Organization.

